# The Roadmap toward Personalized Medicine: Challenges and Opportunities

**DOI:** 10.3390/jpm14060546

**Published:** 2024-05-21

**Authors:** Caterina Cinti, Maria Giovanna Trivella, Michael Joulie, Hussein Ayoub, Monika Frenzel

**Affiliations:** 1 National Research Council (CNR), 00185 Rome, Italy; 2 Agence Nationale de la Recherche (ANR), 75013 Paris, Francemonika.frenzel@agencerecherche.fr (M.F.)

**Keywords:** personalized medicine, ICPerMed, healthcare, health data, precision medicine

## Abstract

In 2019, the International Consortium for Personalised Medicine (ICPerMed) developed a vision on how the use of personalized medicine (PM) approaches will promote “next-generation” medicine in 2030 more firmly centered on the individual’s personal characteristics, leading to improved health outcomes within sustainable healthcare systems through research, development, innovation, and implementation for the benefit of patients, citizens, and society. Nevertheless, there are significant hurdles that healthcare professionals, researchers, policy makers, and patients must overcome to implement PM. The ICPerMed aims to provide recommendations to increase stakeholders’ awareness on actionable measures to be implemented for the realization of PM. Starting with best practice examples of PM together with consultation of experts and stakeholders, a careful analysis that underlined hurdles, opportunities, recommendations, and information, aiming at developing knowledge on the requirements for PM implementation in healthcare practices, has been provided. A pragmatic roadmap has been defined for PM integration into healthcare systems, suggesting actions to overcome existing barriers and harness the potential of PM for improved health outcomes. In fact, to facilitate the adoption of PM by diverse stakeholders, it is mandatory to have a comprehensive set of resources tailored to stakeholder needs in critical areas of PM. These include engagement strategies, collaboration frameworks, infrastructure development, education and training programs, ethical considerations, resource allocation guidelines, regulatory compliance, and data management and privacy.

## 1. Introduction

Personalized medicine perspectives have been recognized years ago as a necessary direction for better therapeutic outcomes [1,2], moving from the concept of basic requirements for clinical use to a translational and regulatory science. Furthermore, personalized medicine as an interdisciplinary topic has become a priority in the research and innovation agenda of the European Commission and national agencies. In November 2016, with the support of the European Commission, the International Consortium for Personalised Medicine (ICPerMed) was launched, involving forty European and international partners, including funding bodies, ministries, and other government structures. The ICPerMed has the general objective of linking the different initiatives related to the development of research projects and implementation of personalized medicine.

In recent years, throughout the increased knowledge of genomics and epigenomics [3] together with biomarkers [4], there has been a flourishing of articles on the applications of personalized medicine in different areas of medicine, especially in oncology [5] and more recently in different and rare diseases [6,7,8]. The use of big data and new technologies, including the use of artificial intelligence, requires many aspects of ethics and equity to be considered [9]. The International Consortium for Personalised Medicine (ICPerMed), through a survey [10] and an active discussion with European and International experts in fields of medical sciences, defined in 2019 a vision for implementing the use of personalized medicine (PM) in 2030, in order to increase effectiveness, economic value, and equitable access for all citizens [11,12]. The discovery of new molecular characteristics and the development of new diagnostic tests, which allow a more detailed redefinition of pathologies as well as the identification of subgroups of patients who respond well to a therapy, opened important scenarios for the medicine of the future.

The PM objectives, applied to public health, are as follows:

o to stratify populations to identify who benefits most from a given treatment;
o to decrease the number of patients treated unnecessarily.

Besides genetic profiles, other important pharmacological characteristics associated with the success of a therapy, such as pharmacokinetic aspects, inter-individual and gender differences, and drug interactions, should be considered. It appears important to avoid the excesses of technical tools in medicine that lead to a depersonalization of the doctor–patient relationship and to unequal access to care.

To face the PM complexity and the multiple scenarios within the ICPerMed organization, a working group (WG2), having a specific activity on “Personalised Medicine in Healthcare”, for three years from 2020 analyzed and defined in some documents the needs and a roadmap for the future [13]. All the critical issues and the necessary actions have been carefully reported.

## 2. Methods

A collection of best practice examples of personalized medicine (PM) with the support of the ICPerMed working groups and the ICPerMed Secretariat took place and are published on the ICPerMed website. The best practice examples published cover different aspects of the value chain. Two different types of best practice examples are considered:

1. The successful translation of PM research into an added value for the patient.
2. Policy making and impact analysis for PM research.

A set of PM application examples were identified, and additional ones were collected based on the suggestions of the ICPerMed members and observers together with the “Best Practice in Personalised Medicine Recognition” activity. The recognition is yearly organized to encourage and disseminate PM implementation as well as to accelerate and maximize the potential impact of the research outcomes and learnings. As an example, the last call was dedicated to a scientific paper that focused on novel approaches for PM implementation, training programs for health personnel, and examples of interdisciplinary or inter-sectoral groups of collaboration (governmental and nongovernmental organizations, academic management, medical research, and Health Care and Industry for the Implementation of PM, including Ethical, Legal, and Social Issues (ELSI) activities).

A panel of experts reviewed the proposals, giving a score based on the following criteria:

1. Knowledge production
2. Research capacity: building and targeting
3. Informing policy and practice
4. Population health and health sector benefits
5. Economic impacts

Additionally, representatives of examples close to implementation or of examples supporting PM implementation were invited to fill in a PM application form and provide further information about their approach [12]. In this way, WG2 revised the collected PM applications and verified their level of implementation before further starting in-depth analysis. A semi-structured data collection form was developed to collect information from representatives of PM application using interviews (individual or group) [13]. A total of nine external stakeholders participated in the interviews. The interview questions were shared with the stakeholders prior to the interviews for information and so that they could be filled in beforehand, to ease the interview flow and the analysis of the discussion outcome afterwards. Interview sessions were recorded upon confirmation of the participants to develop the analysis.

In summary, through a review of PM examples and by dedicated interviews of international and European experts in PM, relevant tasks in PM implementation were identified and the importance of various stakeholder involvement processes underlined.

## 3. Results

**Figure 1:**
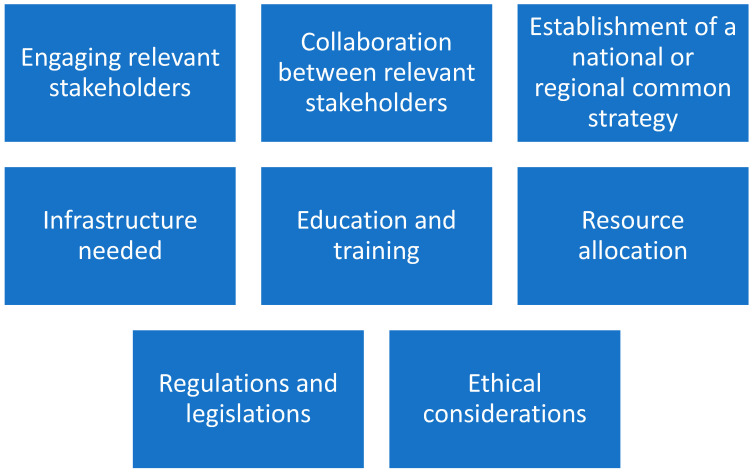
Eight major facilitators in the implementation process of personalized medicine.

By the above methodology, major facilitators have been identified (Figure 1).

It appears important to clarify the facilitators in detail.

The diverse groups of relevant stakeholders include patients, healthcare providers, researchers, policy makers, and industry partners. Their engagement can help to guarantee that the needs and concerns of all stakeholders are considered and that there is strong support for the implementation. PM often requires collaboration and partnerships across sectors and disciplines, by including collaborations between healthcare providers and researchers, as well as partnerships with industry and other organizations. Furthermore, PM often requires a robust infrastructure, including diagnostics and testing technologies, digital health tools, data analytics capabilities, secure data storage and sharing systems, etc. Ensuring that the necessary infrastructure is in place is essential for successful PM implementation. It is important to ensure that healthcare providers and other stakeholders have the necessary knowledge, skills, and resources to implement PM effectively, by requiring training on new technologies, approaches, and tools, as well as education on the ethical and legal considerations. Its implementation may require significant financial and human resources and it is mandatory to monitor and control how these resources will be allocated and managed to ensure effective PM implementation.

Moreover, PM may be a new or unfamiliar concept to many people, and it is important to consider how to effectively communicate about it and address any concerns or misinformation making it accessible to everyone. This may involve engaging with researchers, healthcare providers, patient advocacy groups, policy makers, and other stakeholders to increase public awareness and PM acceptance.

Relative to data, it is important to underline that PM relies on the availability of high-quality data, including clinical and genetic data. Ensuring the privacy and security of this data, as well as addressing any barriers to data sharing, is essential for successful PM implementation.

In summary, engaging the different stakeholders requires a strong collaboration ab initio of PM programs by formally determining governance structures. A clear definition of roles and responsibilities within these collaborative structures is essential to ensure effective coordination and decision making. Moreover, collaborations between public and private sectors, academia, and healthcare institutions are necessary for improving PM research and its realization. Establishing a national governance committee among “healthcare providers, research institutions, policy makers, healthcare professionals, patient advocacy groups, and other key stakeholders” is a pillar to define a common strategy and PM implementation agenda. It is necessary to maintain an appropriate degree of flexibility due to the presence of different health systems and resources at regional and local levels.

In the field of infrastructures, it appears important for PM implementation to organize Information Technology infrastructures, as well as biobanks and genomic and molecular diagnostics. Finally, digital platforms are mandatory for patient education and participation (informed consent, participation in the treatments, and feedback on treatment outcomes).

Education and training programs must include all relevant stakeholders, healthcare professionals, researchers, patients, and the public. It appears crucial to integrate PM topics into the medical and healthcare professionals’ curricula. An extensive collaboration across multiple disciplines is required to conduct research in PM, by organizing teams of experts in genomics, bioinformatics, epidemiology, clinical medicine, statistics, health economics, or implementation science. For the complexity and the differences among “languages” of different fields, it appears important that researchers should be trained to collaborate successfully in interdisciplinary teams. Finally, a specific attention within training programs should be devoted to ethical and legal issues (informed consent, privacy, data sharing…).

A lack or a short supply of financial resources can impede the smooth flow and integration of research outputs and data into practice. Without adequate financial resources, healthcare systems may have big problems when trying to establish strong and sustainable infrastructures, interoperability, and data- or sample-sharing mechanisms and required actions, impeding the effective utilization of patient information and research results for personalized care. Often research funds are limited to specific projects within a strict period with a lack of follow up, which is very important for tools revision derived from clinical outcomes.

Regulations and legislations play an essential role in PM implementation, particularly in relation to the collection, storage, and analysis of patient data. However, the regulations can be complex and fragmented, involving several jurisdictions, agencies, and legal frameworks. To overcome these complexities, it is important to establish clear, interlinked, and adaptable rules to provide guidance on various aspects of PM, i.e., data privacy, consent, research ethics, reimbursement, and clinical implementation. Harmonizing regulations across jurisdictions and disciplines is essential to facilitate innovation and collaboration in the field.

As underlined before, high-qualified personnel with a deep understanding of medical, technical, and scientific regulatory requirements are needed. Nevertheless, recruiting such individuals can be difficult, as there may be a shortage of professionals with the necessary expertise. Additionally, the inclusion of personnel with a data science background is important to leverage the potential of data-driven approaches in PM, but these profiles may still be lacking in healthcare practice.

Finally, the need for ethical considerations has been identified. The ethical field and its knowledge are fundamental to ensuring that PM is developed and applied in an equitable manner, respecting individual rights, and socially responsible. It appears important for the future to pay attention to disparities in PM access, by ensuring that PM technologies and treatments could be accessible to all segments of the population, regardless of socioeconomic status.

Furthermore, some identified critical issues are represented in Table 1.

## 4. Discussion

While a comprehensive strategy for the implementation of PM is outlined before these results, the ICPerMed also recognizes several critical issues that warrant careful consideration, as summarized in Table 1 and reported within the document “Challenges, Opportunities, and Facilitators in Implementing Personalised Medicine” [13]. It has defined that a major challenge is the digital divide, particularly in the context of an aging population that may find it difficult to use devices and engage with telemedicine. Strategies to enhance digital literacy and provide user-friendly technologies are essential to ensure that the benefits of PM and telemedicine are accessible to all age groups. The diverse cultural backgrounds of citizens must be considered in the development and implementation of PM. This includes respecting cultural norms, beliefs, and practices in healthcare delivery and patient engagement strategies. The requirements for implementing PM and novel technologies such as telemedicine differ significantly between urban and rural areas. In rural areas, challenges such as lower population density and limited mobile phone connectivity need specific strategies tailored to their unique context. The relationship between family doctors and citizens varies across different territorial healthcare systems. Strengthening this relationship is crucial, especially in the decentralized model of PM, where family doctors often play a key role in coordinating patients’ care. The high turnover of healthcare professionals and the shortage of doctors, specialists, and nurses pose significant challenges to healthcare systems. Addressing these issues is critical for the sustainable implementation of PM. A holistic approach to healthcare is essential, where healthcare professionals consider the overall well-being of the individual, including psychological support. This approach is intricately linked with the education and training of healthcare professionals, laboratory technicians, IT specialists, as well as patients and citizens. Ensuring that all stakeholders have a broad understanding of the various facets of healthcare, including the psychosocial aspects, is pivotal in PM. Considering the diversity of healthcare systems in Europe or even on a regional level within one country, not one single implementation approach is applicable for all nor could a recommendation be developed for each individual [14]. The harmonization or alignment of healthcare systems could be a solution but would require a complex and long development and implementation process. Therefore, today cross-border collaboration on all levels is essential to provide, by respecting the diversity of the current healthcare systems, access to new technologies, tools, and care to all European citizens.

The COVID-19 pandemic strongly suggested to researchers that sharing data is a fundamental way to proceed with PM [15]. The European Commission (EC) is already approaching the issues relative to the European health data, especially after the COVID-19 pandemic. To release the full potential of health data, the European Parliament is presenting a regulation to establish the European Health Data Space [16]. It has stated that “The European Health Data Space is a health specific ecosystem comprised of rules, common standards and practices, infrastructures and a governance framework that aims at empowering individuals through increased digital access to and control of their electronic personal health data, at national level and EU-wide, and support to their free movement, as well as fostering a genuine single market for electronic health record systems, relevant medical devices and high risk Artificial Intelligence (AI) systems (primary use of data) providing a consistent, trustworthy and efficient set-up for the use of health data for research, innovation, policy-making and regulatory activities (secondary use of data)”. The EC has been actively organizing meetings and preparing documents for the European Health Data Space. A key objective is to maximize the benefits derived from a safe and secure exchange, use, and reuse of health data within the European Union [16]. The European Health Data Space appears as a key pillar of the strong European Health Union, and it is the first common EU data space in a specific area to emerge from the European strategy for data.

Furthermore, many regional projects as well as the European Consortium and Clusters are approaching the issues related to the sharing of data, including safety and security, the use of AI and the associated ethical issues, as well as the active involvement of patients’ associations in PM implementation. As examples it seems important to mention the NAGEN 1000 [17] and ProCAncer-I [18] projects.

At the workshop of the ICPerMed (Pamplona Navarra 17–18 January 2023), Gonzalo Rodríguez, a representative of the Navarra Health Cluster, presented the PM plan in the region and in connection with other regions and national levels. The NAGEN program started in 2020, with a strategic initiative to implement personalized precision medicine until 2030. Electronic data were available from the late 1990s. Emerging from research projects funded by the Department for Industry of the Government of Navarra, the Genomic Medicine Unit brings together its own staff (director, genetic advisor, geneticist, and lab technician), clinical experts from the Navarra Hospital Complex (coordinators from twenty medical specialties), and staff from other Units (Bioinformatics) and Platforms, as well as advisors. As part of NAGEN program, the Genome 1000 Navarra (NAGEN 1000) project is a model for personalized medicine and genomic individuality implementation in the Public Health Service, assuring a multidisciplinary participation of professionals and patients [17].

ProCAancer-I proposed as its objective the organization of an AI platform integrating imaging data and models supporting precision care through prostate cancer’s continuum. In Europe, prostate cancer (PC) is the second most frequent type of cancer in men and the third most lethal. Current clinical practices lead to overdiagnosis and overtreatment, necessitating more effective tools for discriminating between aggressive and non-aggressive disease. The EU-funded ProCAncer-I project proposes to develop advanced artificial intelligence (AI) models to address unmet clinical needs: diagnosis, metastases detection, and prediction of response to treatment [18]. To achieve this, partners will generate a large interoperable repository of health images, and a scalable high-performance computing platform hosting the largest collection of PC Magnetic Resonance Images, used for developing robust PC AI models. To ensure the rapid clinical implementation of the models developed, the project’s partners will robustly monitor performance, accuracy, and reproducibility. From the publication’s list it is possible to recognize all the complexity aspects afforded within the project consortium and the multidisciplinary teams.

To complete the analysis, in terms of fund allocation, it is important to mention the publication of Nardini et al., where it is reported all the European regions that have included personalized medicine as investment priority [14].

## 5. Conclusions

In conclusion, this ICPerMed document, “Challenges, Opportunities, and Facilitators in Implementing Personalised Medicine”, presents a roadmap for the integration of PM into healthcare. Its recommendations offer actionable strategies to overcome existing barriers and harness the potential of PM for improved health outcomes. The success of this endeavor hinges on collaborative efforts, innovative thinking, and a steadfast commitment to ethical principles and common goals but also on the availability of sustainable investments in research, innovation, and healthcare infrastructures. As we advance, it is imperative that all stakeholders, including healthcare professionals, researchers, policy makers, and patients, engage actively in this transformative journey. By doing so, we can unlock the full potential of PM, making it a cornerstone of modern healthcare that is accessible, efficient, equitable, and tailored to the individual needs of patients and all citizens. This paradigm shift, while challenging, holds the promise of a future where healthcare is more precise, predictive, and patient-centered, ultimately leading to better health outcomes and quality of life for all.

## Figures and Tables

**Table 1:** Critical issues for personalized medicine accessibility.

Critical Issues for Personalized Medicine Accessibility	Underlying Healthcare Systems Challenges	Stakeholders
Challenges created by the digital divide, in the context of a progressively aging population	Health inequalities and digital health integration	Home and community care services, patient advocacy groups, public health authorities, technology and telehealth providers
Cultural differences	Health inequalities	Healthcare providers, policy makers, healthcare regulators
Public awareness and acceptance	People empowerment and self-management	Researchers, healthcare providers, patient advocacy groups
Territories with different densities (urban vs. country population) have different needs for implementation (e.g., mobile phone connection and telemedicine organization)	Health and care access and digital health integration	Healthcare providers, government authorities, healthcare regulators, policy makers, patient advocacy groups, technology providers, telecommunication companies
Family doctor and citizen relationship when the family doctor exists *	Care continuity	
Attracting and retaining healthcare professionals	Health workforce	Government and healthcare regulators, healthcare managers, healthcare workers, educational institutions, technology and digital health companies
Interdisciplinary group to approach different medical fields	Collaboration toward personalized care delivery	Researchers, healthcare providers, patient advocacy groups, technology and digital health companies
Holistic approach of healthcare professionals for a single citizen **	Integrated care	Health, well-being, and social care services, technology and telehealth providers, patient advocacy groups

* Not in all health system organizations. ** Strongly connected with the education of Healthcare Professionals, Laboratory Technicians and Information Technology Specialists, and Patients and Citizens. There is a lack of willingness to move from common practice to a patient-centric approach.

## Data Availability

This study did not report any data.
